# Rosetta FlexPepDock to predict peptide-MHC binding: An approach for non-canonical amino acids

**DOI:** 10.1371/journal.pone.0275759

**Published:** 2022-12-13

**Authors:** Nathaniel Bloodworth, Natália Ruggeri Barbaro, Rocco Moretti, David G. Harrison, Jens Meiler

**Affiliations:** 1 Division of Clinical Pharmacology, Department of Medicine, Vanderbilt University Medical Center, Nashville, Tennessee, United States of America; 2 Department of Chemistry and Center for Structural Biology, Vanderbilt University, Nashville, Tennessee, United States of America; 3 Department of Molecular Physiology and Biophysics, Vanderbilt University, Nashville, Tennessee, United States of America; 4 Institute for Drug Discovery, Leipzig University, Leipzig, Germany; Weizmann Institute of Science, ISRAEL

## Abstract

Computation methods that predict the binding of peptides to MHC-I are important tools for screening and identifying immunogenic antigens and have the potential to accelerate vaccine and drug development. However, most available tools are sequence-based and optimized only for peptides containing the twenty canonical amino acids. This omits a large number of peptides containing non-canonical amino acids (NCAA), or residues that undergo varied post-translational modifications such as glycosylation or phosphorylation. These modifications fundamentally alter peptide immunogenicity. Similarly, existing structure-based methods are biased towards canonical peptide backbone structures, which may or may not be preserved when NCAAs are present. Rosetta FlexPepDock *ab-initio* is a structure-based computational protocol able to evaluate peptide-receptor interaction where no prior information of the peptide backbone is known. We benchmarked FlexPepDock *ab-initio* for docking canonical peptides to MHC-I, and illustrate for the first time the method’s ability to accurately model MHC-I bound epitopes containing NCAAs. FlexPepDock *ab-initio* protocol was able to recapitulate near-native structures (≤1.5Å) in the top lowest-energy models for 20 out of 25 cases in our initial benchmark. Using known experimental binding affinities of twenty peptides derived from an influenza-derived peptide, we showed that FlexPepDock protocol is able to predict relative binding affinity as Rosetta energies correlate well with experimental values (r = 0.59, p = 0.006). ROC analysis revealed 80% true positive and a 40% false positive rate, with a prediction power of 93%. Finally, we demonstrate the protocol’s ability to accurately recapitulate HLA-A*02:01 bound phosphopeptide backbone structures and relative binding affinity changes, the theoretical structure of the lymphocytic choriomeningitis derived glycosylated peptide GP392 bound to MHC-I H-2D^b^, and isolevuglandin-adducted peptides. The ability to use non-canonical amino acids in the Rosetta FlexPepDock protocol may provide useful insight into critical amino acid positions where the post-translational modification modulates immunologic responses.

## Introduction

The class 1 Major Histocompatibility Complex (MHC-I), termed the Human Leukocyte Antigen (HLA) in humans, plays a critical role in immune surveillance by enabling the presentation of peptides to patrolling cytotoxic T lymphocytes (CTLs). In every cell of the body, intracellular proteins are hydrolyzed and denatured, and a small fraction of the resulting eight- to eleven-amino acid peptides are loaded onto MHC-I and shuttled to the cell surface, allowing cytotoxic lymphocytes and natural killer (NK) cells to interrogate the intracellular content. If the resulting MHC-I/peptide complex is recognized as “self”, CTLs and NK cells are not activated. Alternatively, if the MHC-I/peptide complex is recognized as non-self, CTL and NK cells are activated to kill the presenting cell [1]. This system has evolved primarily for eliminating viral-infected or malignant cells, but aberrant recognition of modified self-proteins can lead to autoimmune disease [2]. Identifying these intracellular antigens thus has broad applicability and represents one of the greatest challenges in immunotherapy.

Computational tools to predict MHC-I and peptide interactions have made it possible to screen and identify specific antigens from a very large sample space. These binding predictions employ both sequence-based and structure-based methods. Sequence-based methods rely on statistical learning from an experimental dataset to generate a predicted binding affinity, while structure-based methods infer the strength of protein-protein interactions from predicted models. Although sequence-based methods perform well when constrained to parameters set by the training dataset [3], structure-based methods provide several unique advantages. Structure-based methods are not necessarily biased by pre-existing data, and are the preferred method when training data is limited or nonexistent. The models produced by structure-based methods can provide valuable insights into TCR binding and resultant immunogenicity [4, 5]. Importantly, structure-based methods have the potential to generalize to arbitrary peptides, including those with amino acid compositions not previously observed. Post-translational modifications of amino acids, such as glycosylation, lipid adduction, or phosphorylation, produce peptides containing non-canonical amino acids (NCAA) and in doing so significantly broadens the epitope repertoire of MHC-I. These NCAA-containing peptides can serve as the source of neo-antigens resulting in autoimmune disease [6], allow viruses to escape immune recognition [7], or act as targets for tumor recognition [8].

Rosetta FlexPepDock *ab-initio* is a structure-based method for docking short, flexible peptides to a receptor where no prior information about the conformation of the peptide backbone is known. It simultaneously and efficiently samples the space of possible peptide backbone conformations and orientations over the receptor surface and is able to recapitulate near-native conformations [9]. Additionally, the method allows for the incorporation of NCAAs when modeling peptides; a recently published extension of FlexPepDock *ab-initio* incorporates 37 electrophilic NCAAs in a benchmark of covalently bound peptide/receptor complexes [10]. FlexPepDock Refinement, a version of FlexPepDock *ab-initio* that generates a high resolution model from a course-grained canonical peptide backbone conformation, was previously benchmarked to effectively predict peptide-MHC-I binding [11]. However, assumption of a canonical peptide backbone may inadvertently exclude unique backbone conformations induced by post-translational modifications.

Here we benchmark FlexPepDock *ab-initio* for docking peptides containing NCAAs into MHC-I. The FlexPepDock-derived protocol was optimized to generate near-native structures with free conformational sampling, minimizing the required constraints. For an initial protocol evaluation, we docked five unique peptides into five different MHC-I templates, assessing the protocol’s capability to recapitulate “near native” peptide backbone structures. Next, we illustrate that the model scores calculated by Rosetta FlexPepDock *ab-initio* correlate well with predicted peptide binding affinities generated by a NetMHCpan, a popular sequence-based prediction method [3]. Finally, we demonstrate the protocol’s ability to recapitulate peptide backbone structure and binding affinity changes for actual and theoretical peptides containing a variety of NCAAs, including phosphopeptides derived from tumor antigens, the well-characterized glycosylated peptide GP392 isolated from lymphocytic choriomeningitis virus, and isolevuglandin adducted peptides (important drivers of inflammation in cardiovascular disease) [12]. The ability to use non-canonical amino acids in the Rosetta FlexPepDock protocol can provide useful insights into critical amino acid positions where the post-translational modification enables or disrupts interactions with receptors.

## Materials and methods

### Template selection and peptide MHC-I crossdocking with FlexPepDock *ab-initio* and FlexPepDock Refinement

We used a crossdocking strategy to benchmark the FlexPepDock protocol. Five different peptide-MHC-I crystal complexes were selected based on diverse sequences and high-resolution experimental structures. The backbone conformations of peptides bound to the murine Db isoform of MHC-I (H-2D^b^) share a high degree of structural similarity [13]; peptides deviating from this “canonical” backbone structure were intentionally chosen for benchmarking. Peptide/MHC-I binding complexes were extracted from the Protein Data Bank (PDB) (search criteria: “MHC-I complexes D-B alpha chain”, “mus musculus”, unique 9-mer peptide sequences, better than 3.0 Å resolution and diverse peptide conformations, yielding 65 structures on October 14, 2020). Peptides were extracted and re-docked to each of the five MHC-I protein structures, resulting in twenty-five structures (a 5x5 docking analysis), a sample size previously sufficient to benchmark FlexPepDock protocols by other authors [9, 14]. Selected MHC-I protein PDB entries included: 6G9Q, 5M02, 3WS3, 1BZ9 and 1JUF. 5JWD, containing GP392 peptide in complex with H-2D^b^ was used for docking of a glycosylated peptide. 6G9Q was also used for as template for docking isoLG-modified peptides. We employed an identical protocol in our benchmark of HLA-A02:01 structures. PDBs for this benchmark include entries 3D25, 3MRK, 5NMH, 5EU5, and 6O4Z. For crossdocking studies with FlexPepDock Refinement, a peptide backbone template was selected based on overall sequence similarity to the desired peptide to be modeled (excluding identical sequences). Residues were mutated using Rosetta’s SimpleThreadingMover, and the structure prepacked prior to decoy generation.

### Rosetta FlexPepDock *ab-initio* protocol

**Fig 1:**
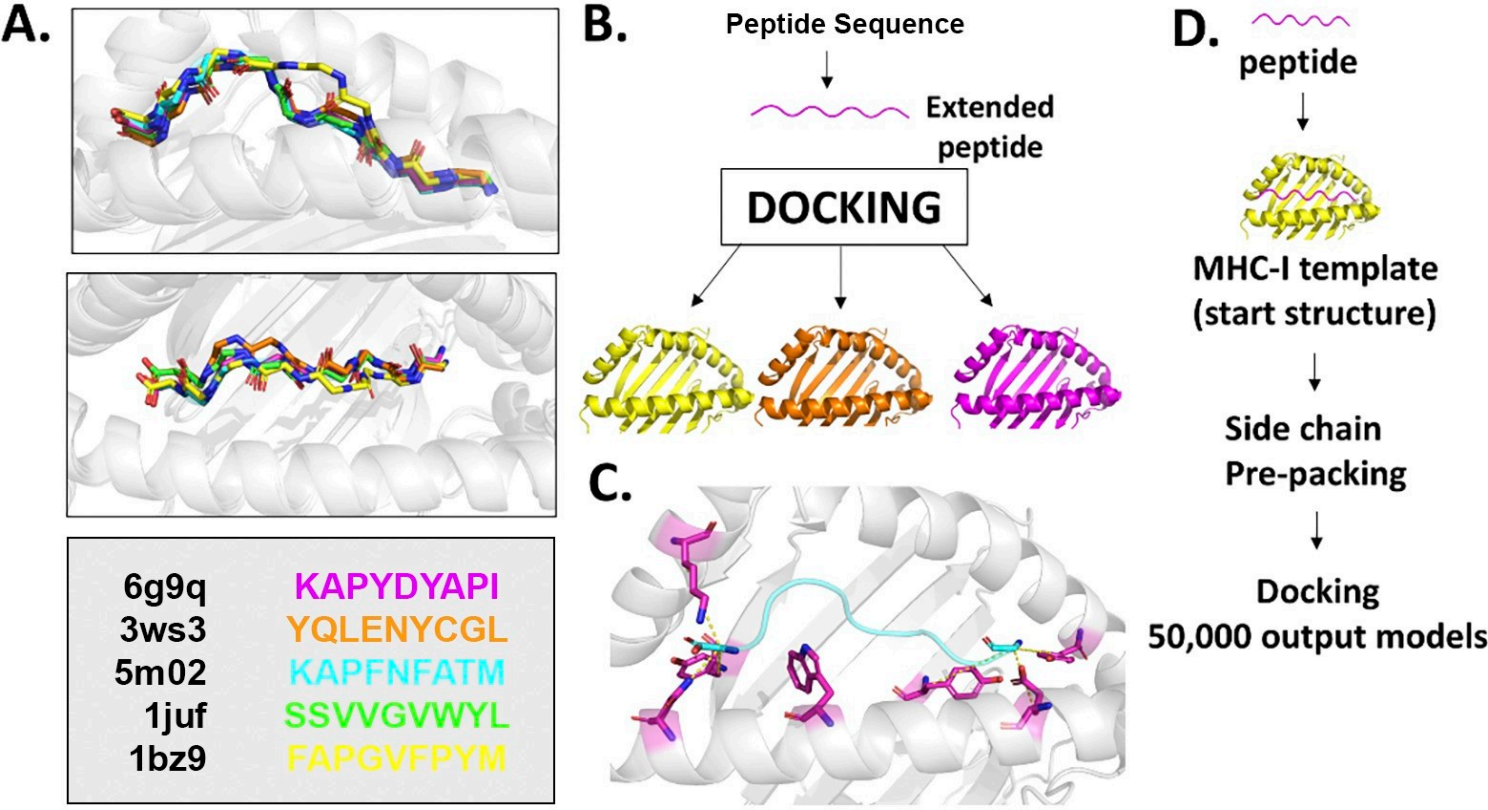
Strategy for docking peptides to MHC-I using FlexPepDock *ab-initio*. (A) Superimposed peptide backbone structures for each of the models used in the initial benchmarking study. (B) Illustrative summary of peptide extension and crossdocking approach. (C) Magenta colored residues illustrate the location of constraints used to define the C- and N- peptide terminus binding pocket as well as the highly conserved location of W73. (D) Illustrative summary of the FlexPepDock *ab-initio* protocol.

The FlexPepDock *ab-initio* protocol implemented in Rosetta-3.12 was performed as previously described [9]. A scheme of the current protocol is shown below in Fig 1, with a full protocol capture in S1 Table in S1 File. Briefly, we performed the following steps:

**Generation of input models**: Peptide chains were removed from MHC-I templates and extended peptide backbone conformations built from FASTA sequences using Rosetta’s BuildPeptide utility. Each of the five peptides was initially positioned close to the peptide binding cleft for each of the five receptor templates, generating twenty-five different complexes (“starting structures”). For RMSD calculations, we kept the native peptide conformation obtained from crystal structure and positioned it at the docking site in each template. We refer to these complexes containing the original peptide conformation as native structures. Peptide/receptor complexes were packed to remove internal clashes and guarantee a uniform conformational background [15, 16].

**Defining constraints**: Rosetta incorporates experimental and structural data to guide model selection in the form of “constraints”, which confer energy penalties to a decoy’s final score if it deviates too far from the defined constraint. In the current peptide-MHC-I docking protocol, we added the “atompair” constraint measuring the distances between atoms in the peptide and receptor (SD ± 0.5Å) to favor models in which the peptide C- and N-terminus (the anchoring residues) were appropriately positioned in the binding cleft. For 1JUF and 1BZ9, additional constraints aimed to fix the position of a highly conserved residue in the receptor (W73) [13] were also included as discussed below. Constraint values used for H-2D^b^ and HLA-A*02:01 are provided in the protocol capture.

**Generating a fragment library for peptides**: We generated a library of 200 trimer and pentamer backbone fragments, which were extracted from experimentally determined protein structures in the Protein Data Bank using the fragment picker application as previously described [17].

**Model generation**: The complete FlexPepDock *ab-initio* protocol (low-resolution modeling and refinement) is detailed by Raveh et al [15]. We generated a total of 50,000 models from each starting structure in parallel batches using the ACCRE CPU cluster. Wall time for each peptide/receptor complex was between 10–16 hours.

**Decoy selection and success metrics**: We used a reweighted score that is a version of Rosetta generic full-atom energy score optimized for peptide-receptor docking, in which interface residues are given double weight, and peptide residues are given triple weight [15, 18]. We defined a docking model as near native if the interface heavy atom backbone atoms of the predicted peptide (bb-RMSD) is ≤ 1.5Å.

### Experimental binding affinities

To evaluate Rosetta energy as a prediction tool, we correlated experimentally determined binding affinities for a sequentially mutated peptide derived from the A/PR/8/34 influenza virus bound to MHC-I H-2D^b^ with energy scores generated by Rosetta FlexPepDock *ab-initio*. For the peptide in question the residue at position 2 was substituted with every possible amino acid and binding affinity subsequently measured [19]. The use of point mutant peptides was particularly important as different residue sequences may have different computational scores and thus may not be comparable [20]. Relative affinities of all twenty possible peptides were displayed as EC50 (nM) and used for Spearman correlations with Rosetta reweighted score as well as theoretical binding affinities generated by NetMHCpan, a popular sequence-based MHC-I binding affinity predictor tool [3]. S2 Table in S1 File shows raw values used in this analysis.

### Parametrization of non-canonical amino acids into Rosetta

Incorporating NCAA for use in Rosetta requires the manual design and optimization of the NCAA side chain structure, generation rotamer library (a collection of possible side chain conformations), and generation of a Rosetta-readable params file [21]. NCAAs were first designed in PyMOL (PyMOL Molecular Graphics System, Version 2.0 Schrödinger, LLC) using available PubChem structures as templates (PubChem CIDs: 24139 and 6267 for glycosylated asparagine, GlcNAC-Asn, and Pubchem CIDs 9548876 and 5962 for isolevuglandin-adducted lysine). PyMOL is a popular molecular graphics program that allows visualization and manipulation of small molecules and proteins in preparation for further downstream applications. Protons and structure angles were fixed using OpenBabel [22]. The resultant molfile was used to generate a params file describing the properties and structure of the NCAA for use in Rosetta. The BCL (Biology and Chemistry Library Project) software suite was used to generate a rotamer or conformer library consisting of 100 side chain conformations as described previously [23].

### Template selection for phosphopeptide docking with FlexPepDock *ab-initio*

In order to demonstrate the extensibility of FlexPepDock *ab-initio* to other MHC-I receptors and NCAA types, we evaluated its performance with phosphopeptides bound to the human MHC-I isoform HLA-A*02:01. For phosphopeptide docking, previously published 9-mer phosphopeptide structures bound to HLA-A*02:01 with experimentally determined binding affinities, available non-phosphorylated counterpart structures, unique sequence, and a resolution better than 3.0 Å were taken from the PDB [8, 24]. The search yielded 8 structures (3QFT, 3QFU, 3FQW, 3FQX, 4NNY, 3BGM, 4NO3, and 4NO5). Peptides were extracted from their corresponding receptor, extended, and re-docked as described in Fig 1D. Constraints for biasing the location of the N- and C- peptide terminus to the HLA-A*02:01 binding pocket were applied as described for MHC-I H-2D^b^, but the constrained residue distances were altered to reflect the average distance of the N- and C- terminus for relevant binding cleft residues in HLA-A*02:01. An additional 3 phosphorylated peptides (and non-phosphorylated counterparts) for which experimentally determined binding affinity was available but which lacked an available structure were also docked to a template HLA-A*02:01 receptor (3FQX) in order to assess predicted binding affinity. Rosetta mean reweighted scores were extracted for the top 0.1% scoring models and compared between phosphorylated and non-phosphorylated peptides after docking to HLA-A*02:01 (Mann-Whitney test).

## Results and discussion

### Addition of constraints defining MHC-I binding pockets and fixing conserved residues improves modeling accuracy

FlexPepDock *ab-initio* is designed to generate models of complexes between flexible peptides and proteins when no prior information about the peptide backbone structure is available. The approach is more generalizable than FlexPepDock Refinement (which refines a peptide structure with an already approximate conformation at the receptor binding site) and thus allows for the generation of unique backbone conformations induced by post-translational modifications [9, 15]. However, the conformational space for such an approach is by necessity much larger. Introduction of biologically relevant, experimentally-determined biases in the form of constraints improves the probability that the correct conformations will be sampled. Additionally, beginning from the same extended peptide backbone conformation avoids biasing the final backbone structure.

**Fig 2:**
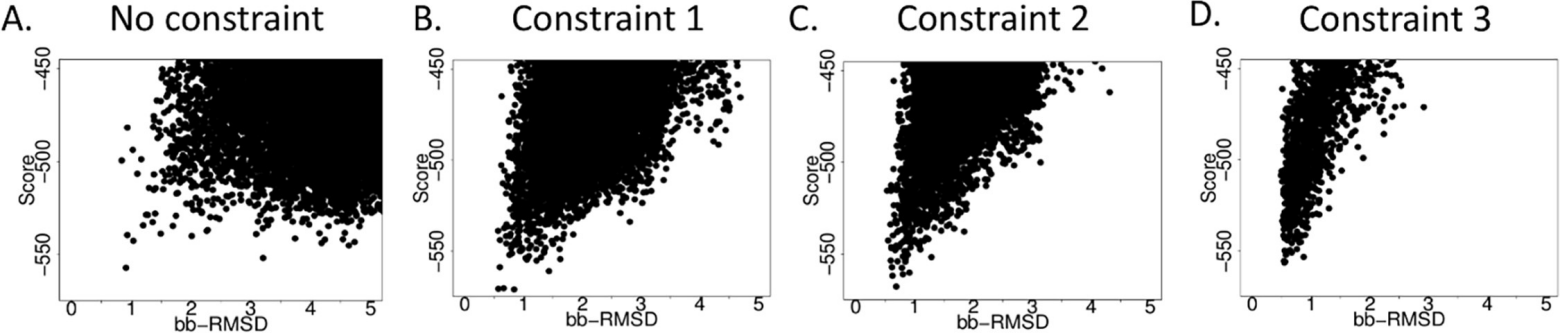
Introduction of constraints designed to recapitulate the known location of the peptide-MHC-I binding pocket and conserved receptor residue W73 improves modeling performance. Plots showing Rosetta reweighted score vs model RMSD for models generated using (A) no constraints, (B) constraint favoring the N- and C- peptide terminus positions in close proximity to the corresponding MHC-I binding pockets, and (C) fixing the position of conserved residue W73 in MHC-I (in addition to the constraints shown in (B)). (D) Modeling performance when canonical peptide backbone dihedral angles alone are used as constraints. Representative data are taken from decoys produced using PDB entry 6G9Q.

In our benchmarking of H-2D^b^ bound peptides we found that omission of constraints produced structures with near-native RMSDs in some but not all peptide/receptor combinations. Modeling performance was improved when we defined the MHC-I binding pocket for the peptide N- and C- terminus (Fig 2A and 2B, constraint 1). This constraint was optimal for most of our benchmark docking cases (6G9Q, 5M02 and 3WS3), generating near-native models (Fig 2B). However, fixing the position of the residue W73 (Fig 2C, constraint 2) improves the docking of 1BZ9 and 1JUF. This constraint, which preserves the relative position of residue W73 in the peptide binding cleft, is specific for the MHC-I Db allele. Class 1 MHC complexes are highly polymorphic and each individual carries a selection of variants; the principle amino acid variations between allelic variants are found at certain sites in the peptide binding cleft, altering receptor peptide interactions. The W73 residue is one of the critical residues that differentiate the MHC-I H-2D^b^ allele from its H-2K^b^ counterpart in mice and HLA-A*02:01 in humans, and its position is highly conserved across structures in the PDB [13]. As result, we used termini restraints plus the W73 constraint for the computational analyses using MHC-I H-2D^b^ as a receptor in this manuscript.

Adding constraints to penalize decoys deviating from canonical backbone dihedral angles for H-2D^b^ bound peptides produces the results with the best RMSD values (Fig 2D, constraint 3). This set of constraints is similar in concept to the FlexPepDock Refinement protocol, which generates a high-resolution refinement from a backbone conformation that is assumed to be the correct approximate configuration. It is not surprising that favoring decoy scores with “correct” dihedral angles would produce near-native decoys; however, the utility of this approach is limited as it assumes the backbone configuration of the peptide in question can be approximated, which, as discussed, may not always be the case (especially if exceptional circumstances such as the inclusion of an unusual NCAA promote an unusual conformation).

### FlexPepDock *ab-initio* accurately recapitulates peptide backbone conformations after docking with MHC-I

Following constraint optimization, we evaluated the ability of FlexPepDock *ab-initio* to recapitulate native MHC-I/peptide complex structures. As shown in Table 1, the protocol recapitulated near-native structures (bb-RMSD ≤ 1.5Å) in the top lowest-energy models for 20 out of 25 docking cases. Two models in the initial benchmark included T cell receptors (TCR) in their original structures which were removed prior to cross-docking (5M02 and 6G9Q). Despite the absence of the TCR, the protocol accurately recapitulated the native backbone structure, and addition of the TCR did not substantially affect the FlexPepDock’s ability to reproduce the peptide backbone structure within 1.5 Å as shown in S1 Fig. Three out of five docking cases in which the top lowest-energy model bb-RMSD was greater than 1.5Å occurred with the docking of peptide 1BZ9 (FAPGVFPYM), and the remaining two occurred with the 1JUF peptide (SSVIGVWYL), peptides that were originally included in our benchmark due to their unique backbone conformation.

**Table 1:** bb-RMSD of top-scoring models generated after docking designated peptides to receptor templates using FlexPepDock *ab-initio*.

Templates	Peptides				
	6G9Q	5M02	3WS3	1BZ9	1JUF
**6G9Q**	0.69	0.88	0.99	1.92	1.22
**5M02**	0.73	0.61	1.48	1.22	1.28
**3WS3**	0.87	1.48	1.01	1.74	1.57
**1BZ9**	0.69	0.76	0.63	1.26	0.99
**1JUF**	0.47	0.92	1.47	1.92	1.60

Previous studies of the 1BZ9 peptide using another structure-based method (AutoDock Vina) also encountered similar modeling difficulties [13]. One possible explanation is that the 1BZ9 epitope is a synthetic peptide, explaining the unusual conformation. The residue at position 5 (P5), usually an anchor residue, fails to bind to the polar pocket, and P6 (Phe) is inserted into cleft changing the pocket conformation [25]. Moreover, when we evaluated the electron density of the crystal structure, we found almost no electron density for the glycine and valine residues of the peptide (P4 and P5). It is likely that the peptide is flexible in this region allowing for multiple conformations. The peptide conformation predicted by FlexPepDock fits the density map about as well as the published structure (S2 Fig), supporting the idea that multiple conformations may be feasible.

The 1JUF peptide (SSVIGVWYL) has a slightly different backbone conformation than usual, in which two water molecules form hydrogen bonds stabilizing the peptide interaction with MHC-I complex at the peptide’s C-terminus [26]. Although this peptide does not use the residue at position 5 as anchor residue, its backbone conformation resembles canonical MHC-I epitopes. While FlexPepDock does not explicitly consider the bridging water interactions which stabilize the crystalized structure, in three out of five docking cases the lowest energy structures had a bb-RMSD ≤ 1.5Å. Even the two predicted structures considered as unsuccessful in the present work are close to the threshold, with bb-RMSD of 1.57Å and 1.60Å, which is considered near native by other authors. This demonstrates that FlexPepDock *ab-initio* is capable of reliably generating structures that could be useful in initial studies where no prior information of the peptide is known.

**Fig 3:**
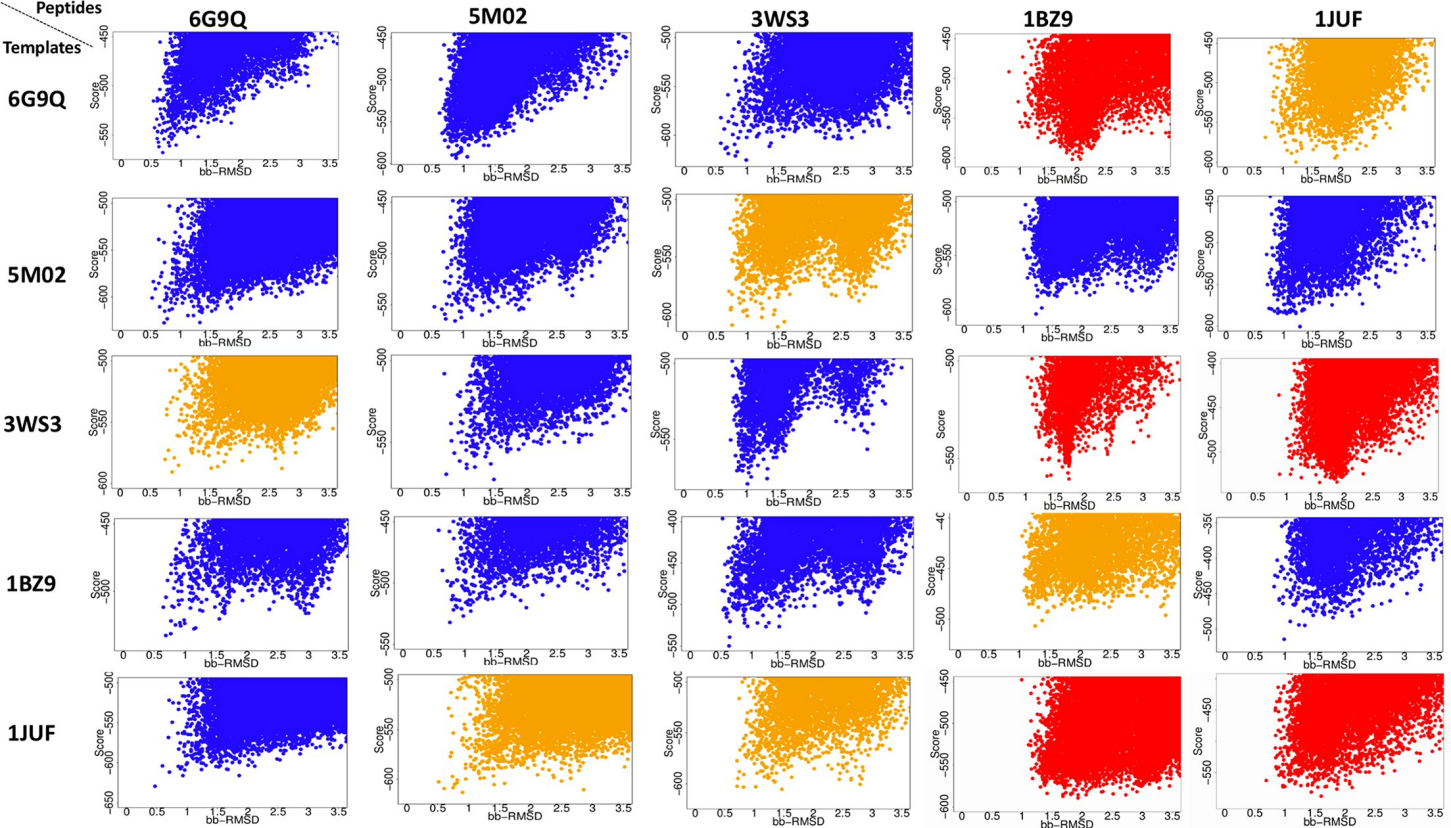
Score vs bb-RMSD funnel plots for each peptide/receptor combination generated in the benchmarking experiment by FlexPepDock *ab-initio*. Plots in blue correspond to production runs in which at least five of the ten lowest energy structures generated had a near-native peptide backbone conformation. Plots in orange indicate that the lowest energy structures generated had more than one conformation. Plots in red represent results where none of the top 10 lowest energy structures had a near-native peptide backbone conformation (≤ 1.5Å).

The representative landscape of models generated by FlexPepDock *ab-initio* for each cross-docking attempt are shown in Fig 3 (see also S3 Fig for a broader spectrum of decoys).

We performed an analogous crossdocking benchmark with five peptides bound to HLA-A*02:01, the human MHC-I allele with the largest number of representative structures in the PDB. HLA-A*02:01 bound peptides have a peptide backbone configuration that, while relatively well conserved, is distinct from peptides bound to H-2D^b^ (S4A Fig). Using literature-derived constraints favoring decoys with correctly positioned N and C-termini and preserving the location of residue W146 (highly conserved in HLA-A*02:01 [13]), crossdocking peptides to HLA-A*02:01 produced decoys with bb-RMSD values ranging from 1.39–2.98 Å (S4A–S4C Fig). The overall performance was notably poorer when compared to *ab-initio*’s predictions for peptides bound to H-2D^b^, with only a handful of benchmarked cases recapitulating peptide backbone structure within 1.5 Å. We repeated the crossdocking study with HLA-A*02:01, this time using FlexPepDock Refinement and a starting peptide backbone structure template selected after aligning and scoring other non-identical HLA-A*02:01 bound peptides available in the PDB (S5A Fig). Results were significantly improved, with near-native (and in some cases sub-angstrom) bb-RMSD’s for nearly every peptide/receptor pair modeled (S5B and S5C Fig). The fact that backbone RMSD values were improved is not surprising given the starting structures were not far removed from the native. What is more notable is the accurate recapitulation of interface residue RMSD, including side chain configuration, which is critical to inferring peptide binding affinity with MHC-I and specificity for any TCRs (S5D Fig).

Taken together our results suggest that selecting appropriate experimental- or literature-derived constraints is critical for generating accurate peptide-MHC-I models using FlexPepDock *ab-initio*. Additionally, performance may vary from one MHC-I allele to another, in no small part to a lack of available information to inform constraint selection. For well-characterized MHC-I alleles where significant deviation from canonical backbone structure is considered unlikely, using FlexPepDock Refinement can circumvent these barriers.

### Reweighted scores generated by FlexPepDock *ab-initio* can predict relative peptide binding affinity for MHC-I

**Fig 4:**
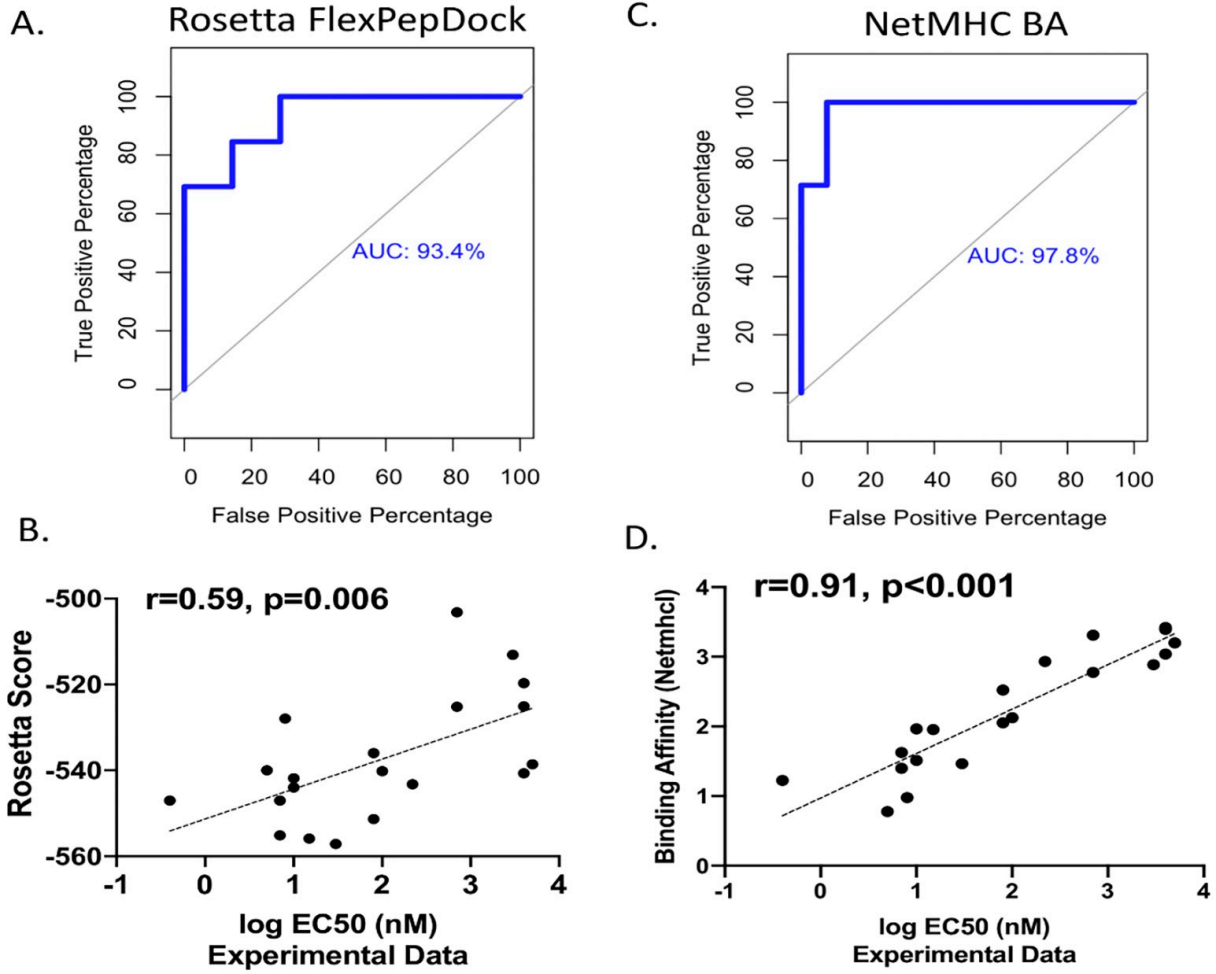
Rosetta FlexPepDock *ab-initio* reweighted score correlates with relative binding affinity and can be used as a binary discriminator to predict a peptide’s ability to bind to MHC-I. Receiver-operator curve for peptides predicted to be binders by (A) FlexPepDock and (C) NetMHCpan. An EC50 of 500nm was used as the cuttoff to distinguish binder from non-binder for experimentally determined binding affinity. (B,D) Correlations between log transformed binding affinity and (B) FlexPepDock reweighted score or (D) predicted binding affinity by NetMHCpan.

We then evaluated the ability of FlexPepDock *ab-initio* to predict peptide binding affinity to MHC-I. For this analysis, we used previously published relative binding affinities of twenty peptides derived from an influenza virus peptide (ASNENMETM), where the authors mutated the residue at position 2 and replaced it by each of the twenty canonical amino acids [19]. We sequentially docked each peptide into a template receptor for MHC-I (6G9Q) with constraints as described. We also compared the experimental binding affinities with predicted binding affinities generated by NetMHCI BA 4.1 pan [3]. ROC analysis revealed 80% true positive rate and 40% false positive rate, achieving a prediction power of 93% as indicated by the area under the curve (AUC; Fig 4A), and Rosetta FlexPepDock energies show a statistically significant correlation with experimentally determined binding affinity (r = 0.59, p = 0.006) (Fig 4B). Our results compare favorably with previously published sequence-based benchmarks using MHC-I templates, where AUC ranged between 55% and 91% [14]. Binding affinities generated by the sequence-based method NetMHCI pan correlated more strongly to their experimental counterparts (r = 0.91 p<0.001) (Fig 4C and 4D; a complete list of peptides and binding affinities can be found in S2 Table in S1 File). It is not entirely unexpected that NetMHCI is able to achieve such strong results, as NetMHCI BA learning tool was trained with available experimental datasets of >1,250 validated T cell epitopes described in the IEDB, with influenza epitopes among them [3]. One important limitation of NetMHCI, as with all sequence-based methods, is that it is unable to evaluate peptides containing non-canonical amino acids, nor can immunogenicity necessarily be inferred from its results as MHC-I binding does not necessarily correlate with T cell responses. One important advantage of structure-based methods, including our approach, is that the predicted conformation can allow further studies of the MHC-I/peptide/T cell receptor ternary complex and can be incorporated with pre-existing structure based methods to assess TCR binding and activation [27].

### FlexPepDock *ab-initio* can generate the structures of peptides with NCAA from a variety of post-translational modifications

**Fig 5:**
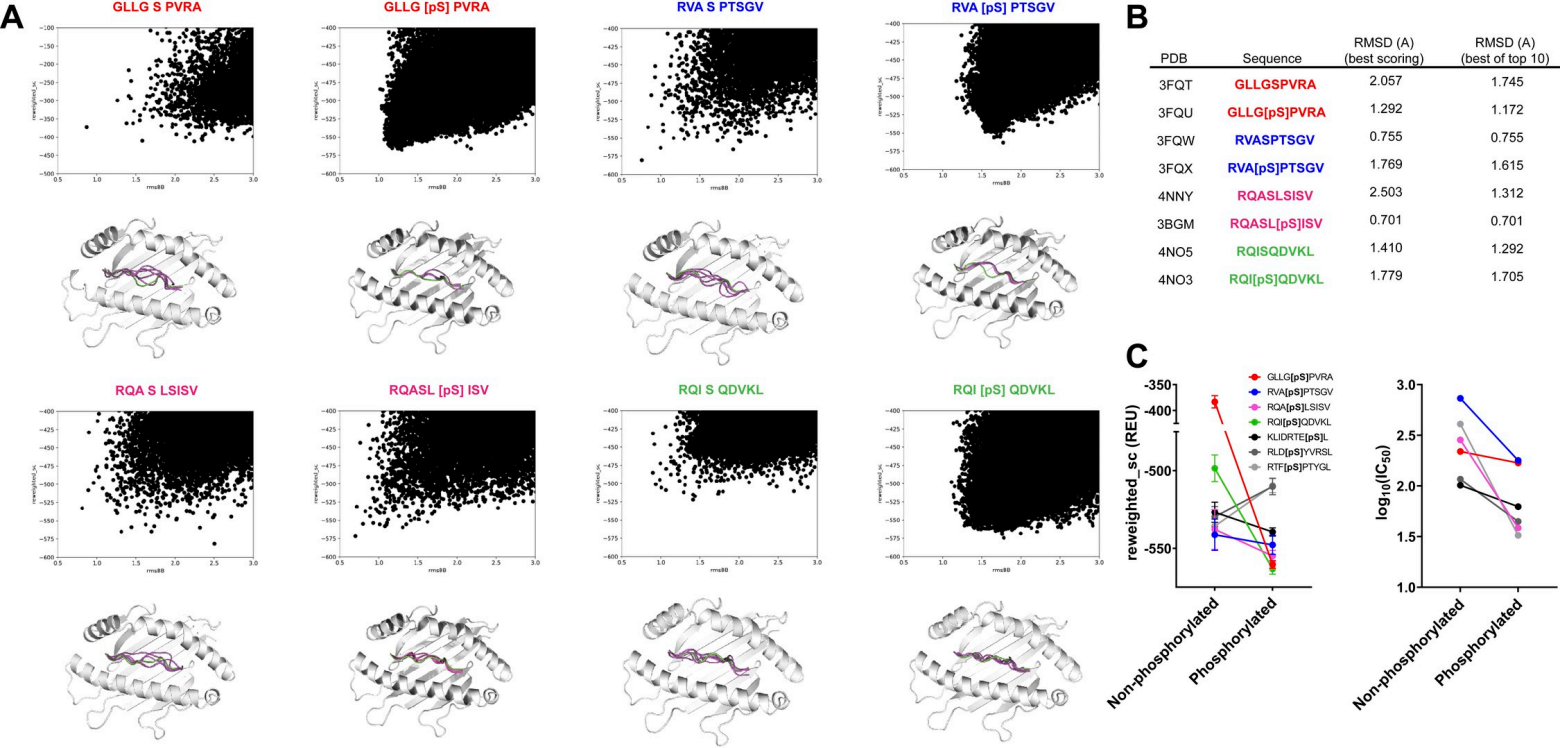
FlexPepDock *ab-initio* can recapitulate the backbone structure of phosphopeptides bound to HLA-A*02:01. (A) Backbone structures for the top 5 scoring decoys generated by FlexPepDock *ab-initio* for phosphorylated peptides and their non-phosphorylated counterparts (phosphorylated residue shown in brackets with a “p” suffix). (B) bb-RMSD values for each peptide for the top-scoring decoy and for an aggregate of the 10-top scoring decoys. (C) Graph showing mean reweighted score (REU = relative energy units) for the top scoring 0.1% of decoys generated (left) and experimental binding affinity (log_10_IC50, right) for each peptide. All differences between unphosphorylated or phosphorylated peptides are noted to be statistically significant (n = 50, p<0.001 by Mann-Whitney test) (a summary of score values and IC50 values for each peptide used in this analysis can be found in S3 Table in S1 File).

We evaluated FlexPepDock *ab-initio*’s ability to recapitulate the structure of peptides with NCAA from various post-translational modifications known to be important in a variety of disease processes, including phosphorylation [8], glycosylation [7], and isolevuglandin-adduction [2]. For phosphopeptides, we selected previously published structures with experimentally determined binding affinity as described in the methods, and docked the peptides to their corresponding HLA-A*02:01 receptor after generating a rotamer library and Rosetta params file for phosphorylated serine as described. Results are shown in Fig 5. For two of four cases for the phosphorylated peptides and two of four cases for their unphosphorylated counterparts, the top structure by score corresponds to a near native structure (< 1.5 Å bb-RMSD). When the top-10 scoring models are aggregated at least one near-native structure is present in all but 3 cases (3FQT, 3fQX, and 4NO3), and of those remaining cases the best bb-RMSD was less than 1.75 (Fig 5A and 5B). We recapitulated these results with FlexPepDock Refinement (S6 Fig) which, consistent with our HLA-A*02:01 benchmark, outperforms *ab-initio* at predicting peptide backbone conformation. For four of the six peptide/phosphopeptide pairs (including those with no available structure but with published binding affinities), FlexPepDock *ab-initio* predicts a statistically significant decrease in mean reweighted score of the top 0.1% of decoys after phosphorylation (though the absolute value of this decrease is relatively small for peptides RVA[pS]PTSGV and KILDRTE[pS]L), consistent with experimental results showing improved binding affinity after phosphorylation [8, 24] (Fig 5C). For one peptide/phosphopeptide with a published structure but without available binding affinities, Rosetta FlexPepDock *ab-initio* predicts a relative increase in binding affinity as well, consistent with the observation and phosphorylation tends to result in greater MHC-I binding avidity [24].

**Fig 6:**
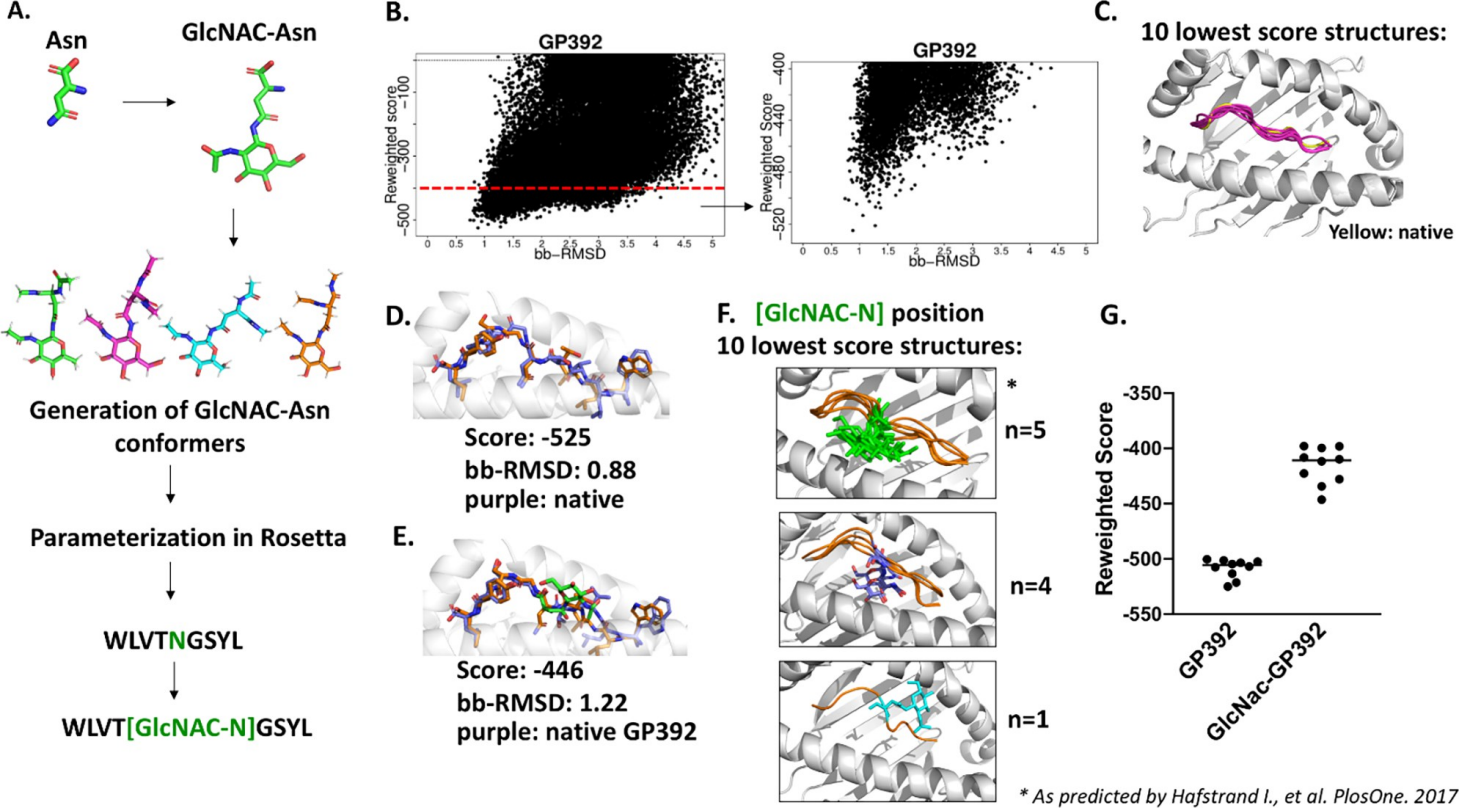
FlexPepDock *ab-initio* recapitulates the native backbone structure of a glycosylated peptide and its unmodified counterpart, and correctly predicts a decrease in binding affinity after glycosylation. (A) Scheme for generating the NCAA for use in Rosetta. (B) Plots of reweighted score values generated by Rosetta and peptide backbone RMSD. (C) Superposition of the top-10 peptide backbones by score, with the native peptide shown in yellow. (D-E) Backbone conformation of the top-scoring models for (D) GP392 and (E) glycosylated GP392 (predicted model in orange, native in purple, and glycosylated residue for the predicted model shown in green). (F) A clustering of glycosylated asparagine side chain positions for the top-10 models by score. (G) The relative binding affinity of GP392 and its glycosylated counterpart, showing that glycosylation is predicted to decrease binding affinity (as assessed by an increase in the reweighted score for the top 10 scoring decoys).

To validate FlexPepDock *ab-initio* with glycosylated epitopes, we used a peptide derived from Lymphocytic choriomeningitis virus (LCMV). LCMV contains a 9-mer antigen with a known glycosylation motif named GP392 (WLVTNGSYL). An N-acetylglucosamine (GlcNAc) sugar moiety binds to the side chain of the asparagine residue within the conserved motif Asn-X-Ser/Thr where X is an amino acid other than Pro [7]. The antigen “GP392” upon glycosylation decreases its binding affinity to H-2D^b^ and presents no cytotoxic response [7]. A structure for the non-glycosylated variant of GP392 in complex with MHC-I H-2D^b^ is available in the PDB illustrating the location of glycogen-binding moiety [28], though no solved structure for the glycosylated epitope exists. Here, we used Rosetta FlexPepDock *ab-initio* to recapitulate both GP392 forms and infer the effects of glycosylation of epitope binding affinity. Our approach is outlined in Fig 6A. Briefly, an asparagine N-linked N-acetylglucosamine (GlcNAc) was designed in PyMOL, with rotamer libraries and Rosetta params files generated as detailed in the methods. FlexPepDock *ab-initio* accurately recapitulated the peptide’s backbone conformation for the non-glycosylated variant (bb-RMSD = 0.88, Fig 6B–6D) and predicted a similar canonical backbone conformation for the glycosylated variant (bb-RMSD = 1.22Å) (Fig 6E). The 10 lowest score structures had bb-RMSD <1.5Å, with three unique GlcNAc conformational groups observed (Fig 6F). Consistent with the observation that glycosylation reduces GP392’s binding affinity for MHC-I H-2D^b^ [7], the top 10 generated models for glycosylated GP392 had on average a higher reweighted score, suggesting a less favorable peptide/receptor interaction (Fig 6G).

**Fig 7:**
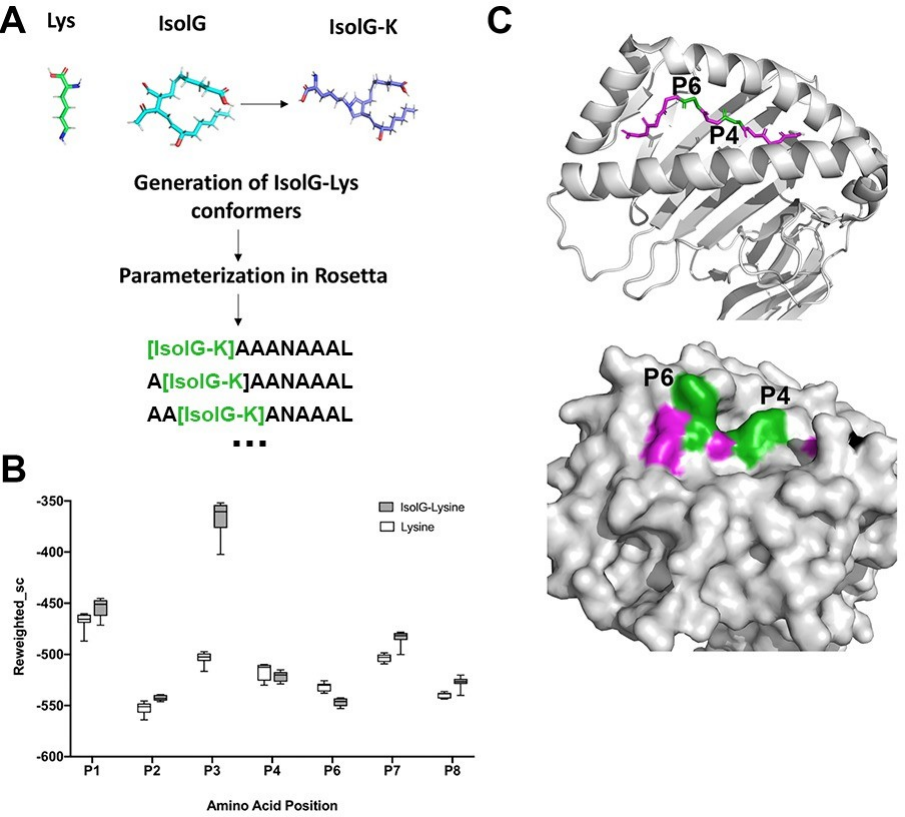
Docking hypothetical isoLG-modified peptides to MHC-I with FlexPepDock *ab-initio* predicts ideal residue locations for isoLG adduction. (A) Scheme for generating the isoLG modified lysine for use in Rosetta, and for sequential modification of a hypothetical lysine-containing peptide. (B) Residues P4 and P6 show a decrease in reweighted score after lysine adduction with isoLG (when compared to lysine-only peptides), suggesting energetically preferential adduction sites. Solid line indicates the mean “reweighted score” value of the top 10 decoys, with the solid box edges corresponding to the 1^st^ and 3^rd^ quartiles and error bars to the minimum and maximum reweighted score values. (C) Diagram showing the prototypical peptide background structure of an epitope bound to MHC-I H-2D^b^ (6G9Q) (cartoon structure shown on top, and surface electron density shown on bottom). Peptide residues are in magenta, and residues P4 and P6 are shown in green.

As an example of this protocol’s applicability to infer data from epitopes for which no analogous starting structures exist, we turned our attention next to isolevuglandin-adducted peptides. Isolevuglandin (isoLG) adduction is a post-translational modification that occurs when oxidized arachidonic acid derivatives covalently bind to lysine residues in proteins. The resultant isoLG-modified protein proteolytically cleaved and presented as an antigen recognized as foreign by the immune system, driving an inflammatory response that contributes to cardiovascular morbidity in a variety of diseases including heart failure and hypertension [29, 30]. While isoLG adducted peptides are a well-described phenomenon in both mice and humans [12], to date the sequence or sequences of residues comprising this family of neoantigens remains undetermined. To infer which residues in an MHC-I presented epitope may favor lysine adduction, we generated a hypothetical peptide with the minimum two key anchor residues [31] for MHC-I H-2D^b^ (asparagine at P5 and leucine at P9) and alanine residues (chosen for their unobtrusive side chain) elsewhere. We next sequentially replaced alanine with lysine or an isoLG modified lysine at P1-P4 and P6-P8 and attempted to dock the resulting peptide to MHC-I H-2D^b^ using Rosetta FlexPepDock *ab-initio*, as shown in Fig 7A. Substitution of lysine for alanine had a minimal effect on peptide backbone configuration (bb-RMSD ≤ 1.5Å when compared to the conformation of AAAANAAL). A complete table of bb-RMSD values is provided in S4 Table in S1 File. For the modified peptides, the replacement of lysine by isoLG-lysine at positions 1, 2, 7 and 8 slightly increased energy scores suggesting that the modification decreased peptide binding affinity (Fig 7B). This increase was most pronounced at position 3. Substitution at positions 4 and 6 improved scores, suggesting that residues with adducted lysine residues restricted to these positions are more likely to be presented by MHC-I H-2D^b^. As shown in Fig 7C, P4 and P6 (shown in green) correspond to regions that extend out of the peptide binding groove for a prototypical MHC-I H-2D^b^ epitope (6G9Q). While this may provide some insight on preferred locations for isoLG adduction, it is also important to note that the observed energy decrease after isoLG adduction at these positions may not necessarily generalize to other peptide sequences, as alternative residue side chains could work to stabilize or destabilize the interaction. That being said, FlexPepDock *ab-initio* provides a versatile tool to perform similar *in silico* studies with various peptide candidates to gain a better understand of how such modifications alter epitope interaction with MHC-I and with T cell receptors.

## Conclusions

In the present work we have illustrated that Rosetta and FlexPepDock *ab-initio* can be used to model peptide-MHC complexes containing NCAA from a variety of post-translational modifications important in disease with a reasonable degree of accuracy, and can be leveraged to provide insights into immunogenicity. Constraints significantly improve its performance but should be optimized for each MHC receptor class. That being said our objective in developing this protocol was not to create a general-use, fully automated peptide-MHC modeling tool (though such a tool would be welcome), but rather to validate that an existing protocol (FlexPepDock *ab-initio* and FlexPepDock Refinement) can be used to accurately predict the structure of NCAA-containing epitopes. Additionally, this approach was evaluated for peptides that are 9 amino acids in length. While this is the most common epitope length presented by class 1 MHC, caution must be undertaken when applying this protocol to peptides of 10-mer length or higher, especially given the enhanced flexibility and ensemble of possible conformations conferred by a longer peptide. Finally, while the approach performs well when tested against available structures, an extensive evaluation of this approach’s efficacy is hampered in no small part by the lack of MHC-1/peptide structures containing NCAA’s available in the PDB. This is unfortunately a barrier that any computational approach will face until additional experimental models are generated, at which point this and other methods should be re-evaluated. That being said, the benchmarking and application of FlexPepDock *ab-initio* and Refinement to study the docking of immunogenic peptides with post-translational modifications to MHC-1 is novel and provides an innovative method for medium-throughput screening of peptides with non-natural amino acids to evaluate MHC-I/peptide and eventual T-Cell receptor interaction.

## Supporting information

S1 Fig FlexPepDock *ab-initio* accurately recapitulates epitope backbone structure for peptides bound to MHC-1 in the presence of a T cell receptor. (A) Funnel plot of backbone RMSD values vs Rosetta FlexPepDock reweighted score values for 6G9Q and 5M02; window defined by the red dotted insert is shown on the bottom to better illustrate the convergence of low-scoring decoys with native structures. (B) Top 5 scoring peptide backbone models (magenta) superimposed on the native peptide (green) and TCR (cyan). (C) Table of RMSD values showing that top scoring models accurately recapitulate the native peptide backbone structure regardless of the presence or absence of the TCR.
(TIF)

S2 Fig The 1BZ9 epitope (yellow) does not have the conventional MHC-I epitope conformation. (A) Superposition of 1BZ9 epitope conformations predicted by FlexPepDock *ab-initio* (magenta), epitope conformation from the deposited crystal structure (yellow) and common pattern epitope conformation into MHC-I (cyan). (B) Electron density map showing almost no electron density for the glycine and valine resides of the peptide 1BZ9.
(TIF)

S3 Fig Plot containing all decoys with reweighted score below 0 for peptides docked with MHC-I templates. (TIF)

S4 Fig FlexPepDock *ab-initio* can recapitulate HLA-A*02:01 bound peptide backbone structures. (A) (Top) representative backbone structures for a peptide bound to H-2D^b^ (green) and HLA-A*02:01 (cyan), illustrating that the two MHC-I receptors prefer distinct peptide conformations. (Bottom) Table of PDB structures and peptide sequences used for the crossdock benchmark. (B) Funnel plots showing the correlation between FlexPepDock *ab-initio* reweighted score values and peptide backbone RMSD. (C) Table of backbone RMSD values for crossdocked peptides.
(TIF)

S5 Fig FlexPepDock Refinement produces peptide conformations with near-native (and in many cases sub-angstrom) accuracy. (A) Overview of template selection process for FlexPepDock Refinement. (B) Table of PDB structures used as templates and their sequences. (C) Table of peptide backbone heavy-atom RMSD values and (D) all interface residue heavy-atom RMSD values for crossdocked peptides.
(TIF)

S6 Fig FlexPepDock Refinement can predict phosphopeptide conformation with near-native accuracy. (A-B) Funnel plots illustrating the relationship between FlexPepDock Refinement reweighted score and peptide backbone RMSD. (C) Top 10 best-scoring structures generated by FlexPepDock Refinement (shown in magenta, native peptide in green). (D) Table of RMSD values for the best scoring and best of the top 10 best scoring decoys produced.
(TIF)

S1 File Contains supporting tables. (DOCX)

## List of abbreviations

CTL: Cytotoxic T-lymphocyte
H-2D^b^: Murine Db isoform of the class 1 major histocompatibility complex
HLA: Human leukocyte antigen
HLA- A*02:01: Human leukocyte antigen allele A2
isoLG: Isolevuglandin
LCMV: Lymphocytic choriomeningitis virus
MHC-I: Major histocompatibility complex, class 1
NCAA: Non-canonical amino acid
NK: Natural killer cell
PDB: Protein Data Bank
bb-RMSD: Backbone heavy atom root-mean-square distance
