# Supplementary figures and images for: Rosetta FlexPepDock to predict peptide-MHC binding: An approach for non-canonical amino acids
Source: PLoS One. 2022 Dec 13;17(12):e0275759. doi: 10.1371/journal.pone.0275759 (PMC9746977; doi:10.1371/journal.pone.0275759)

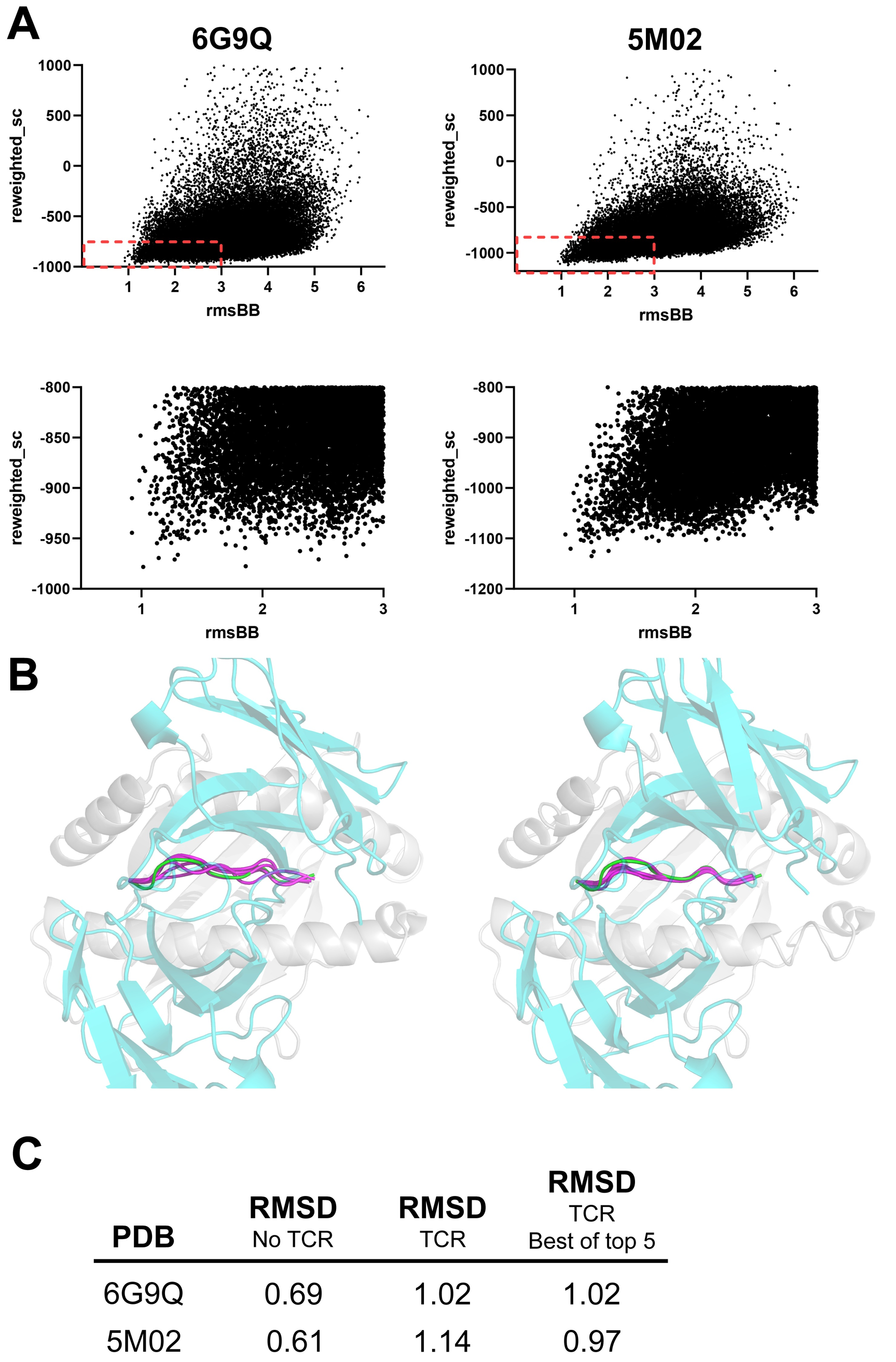

Supplement: S1 Fig — (A) Funnel plot of backbone RMSD values vs Rosetta FlexPepDock reweighted score values for 6G9Q and 5M02; window defined by the red dotted insert is shown on the bottom to better illustrate the convergence of low-scoring decoys with native structures. (B) Top 5 scoring peptide backbone models (magenta) superimposed on the native peptide (green) and TCR (cyan). (C) Table of RMSD values showing that top scoring models accurately recapitulate the native peptide backbone structure regardless of the presence or absence of the TCR. (TIF) [file pone.0275759.s001.tif]

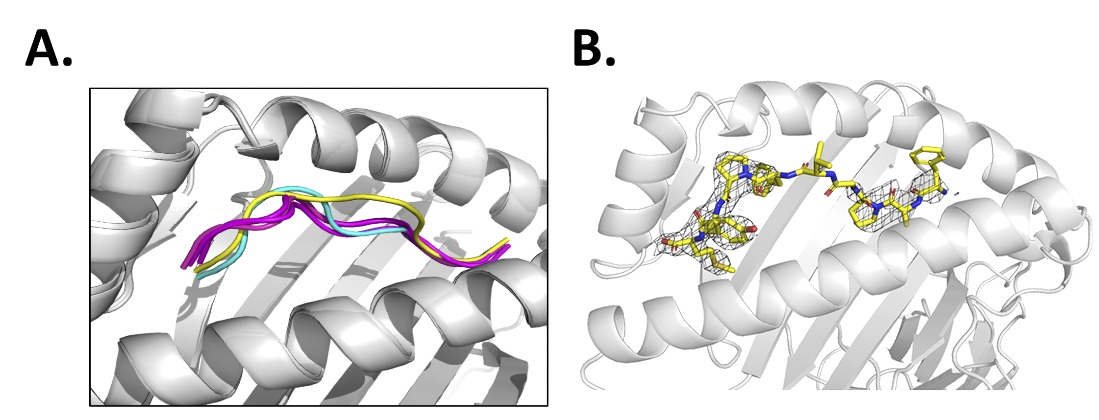

Supplement: S2 Fig — (A) Superposition of 1BZ9 epitope conformations predicted by FlexPepDock ab-initio (magenta), epitope conformation from the deposited crystal structure (yellow) and common pattern epitope conformation into MHC-I (cyan). (B) Electron density map showing almost no electron density for the glycine and valine resides of the peptide 1BZ9. (TIF) [file pone.0275759.s002.tif]

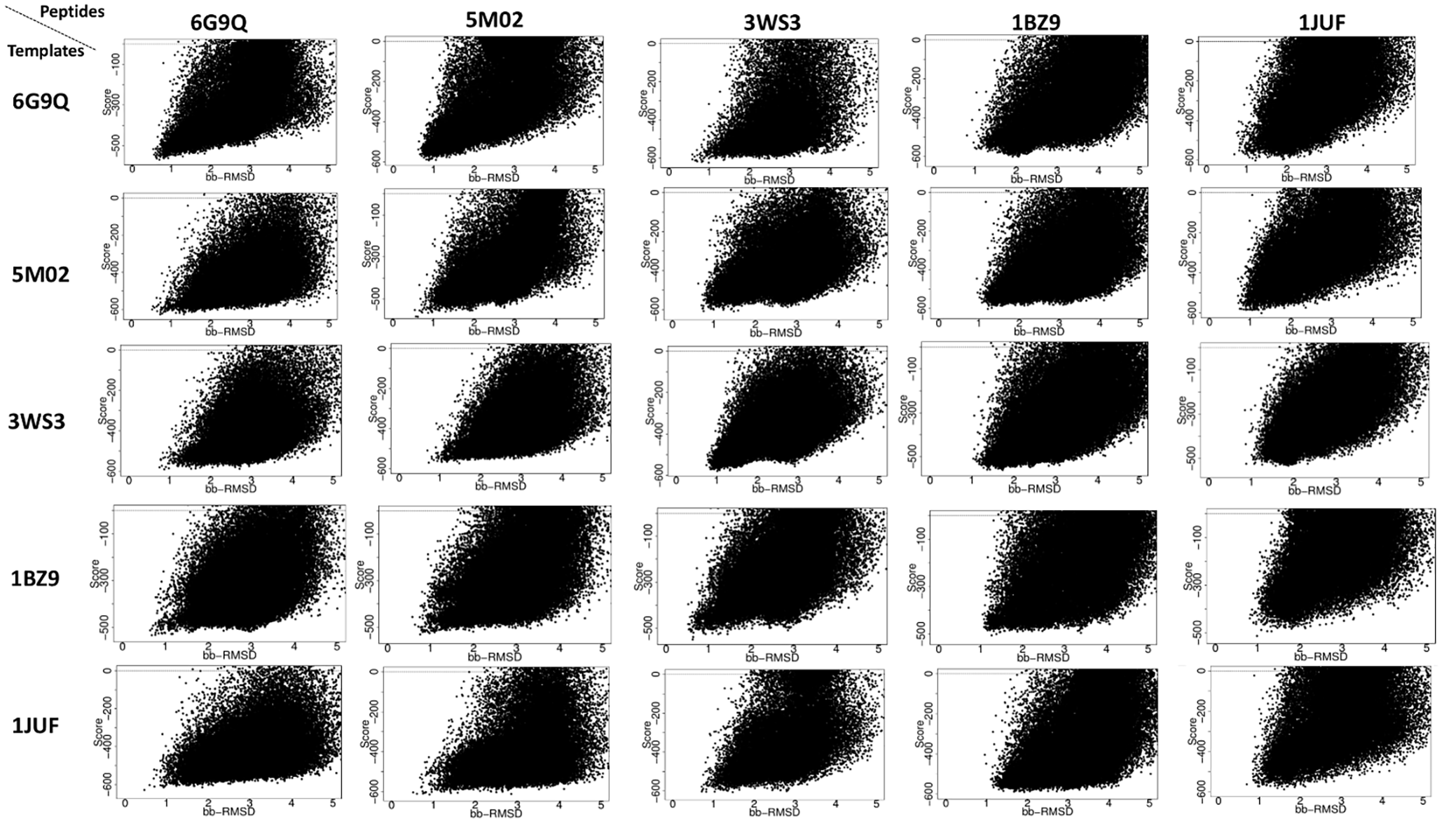

Supplement: S3 Fig — (TIF) [file pone.0275759.s003.tif]

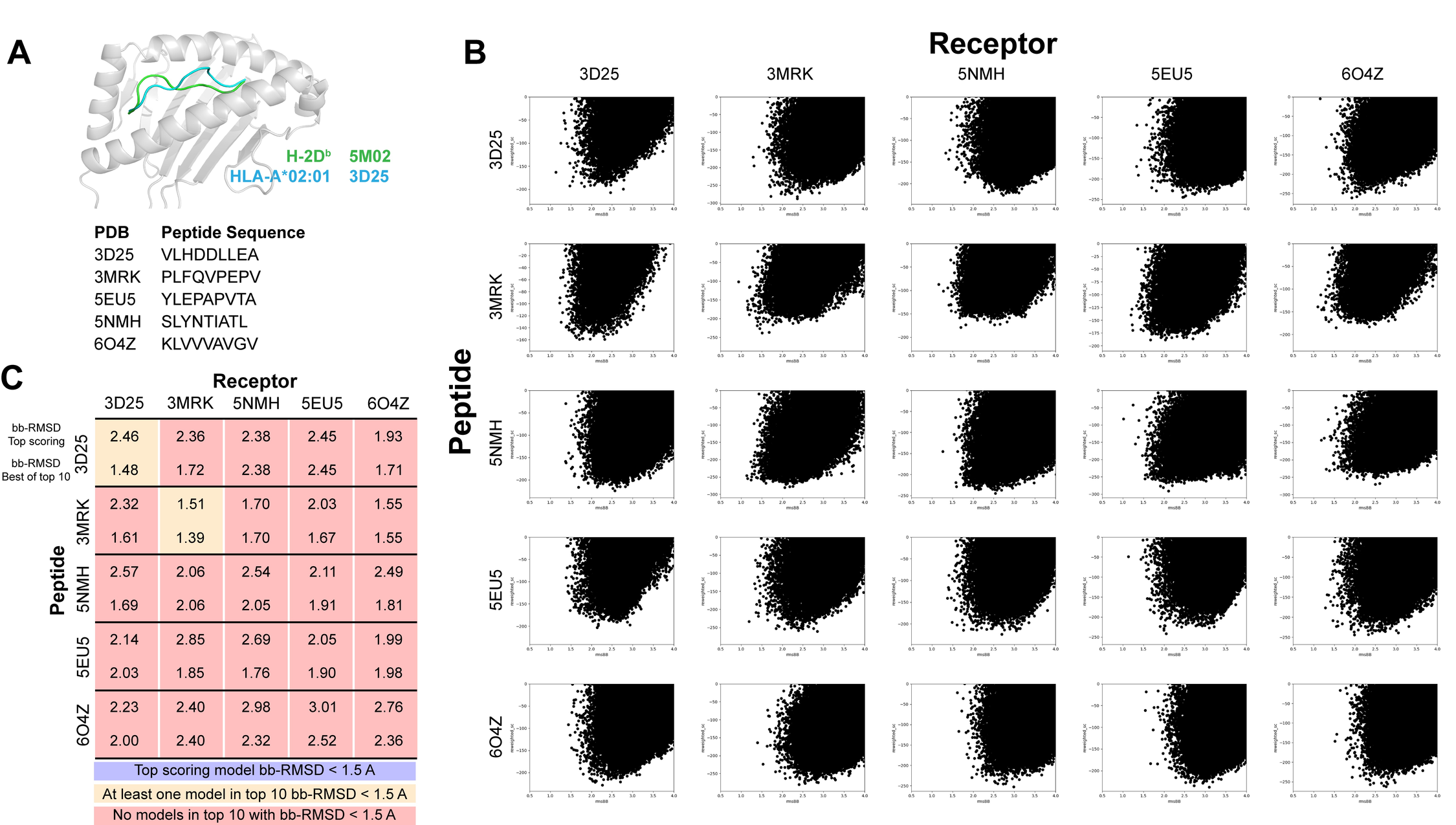

Supplement: S4 Fig — (A) (Top) representative backbone structures for a peptide bound to H-2Db (green) and HLA-A*02:01 (cyan), illustrating that the two MHC-I receptors prefer distinct peptide conformations. (Bottom) Table of PDB structures and peptide sequences used for the crossdock benchmark. (B) Funnel plots showing the correlation between FlexPepDock ab-initio reweighted score values and peptide backbone RMSD. (C) Table of backbone RMSD values for crossdocked peptides. (TIF) [file pone.0275759.s004.tif]

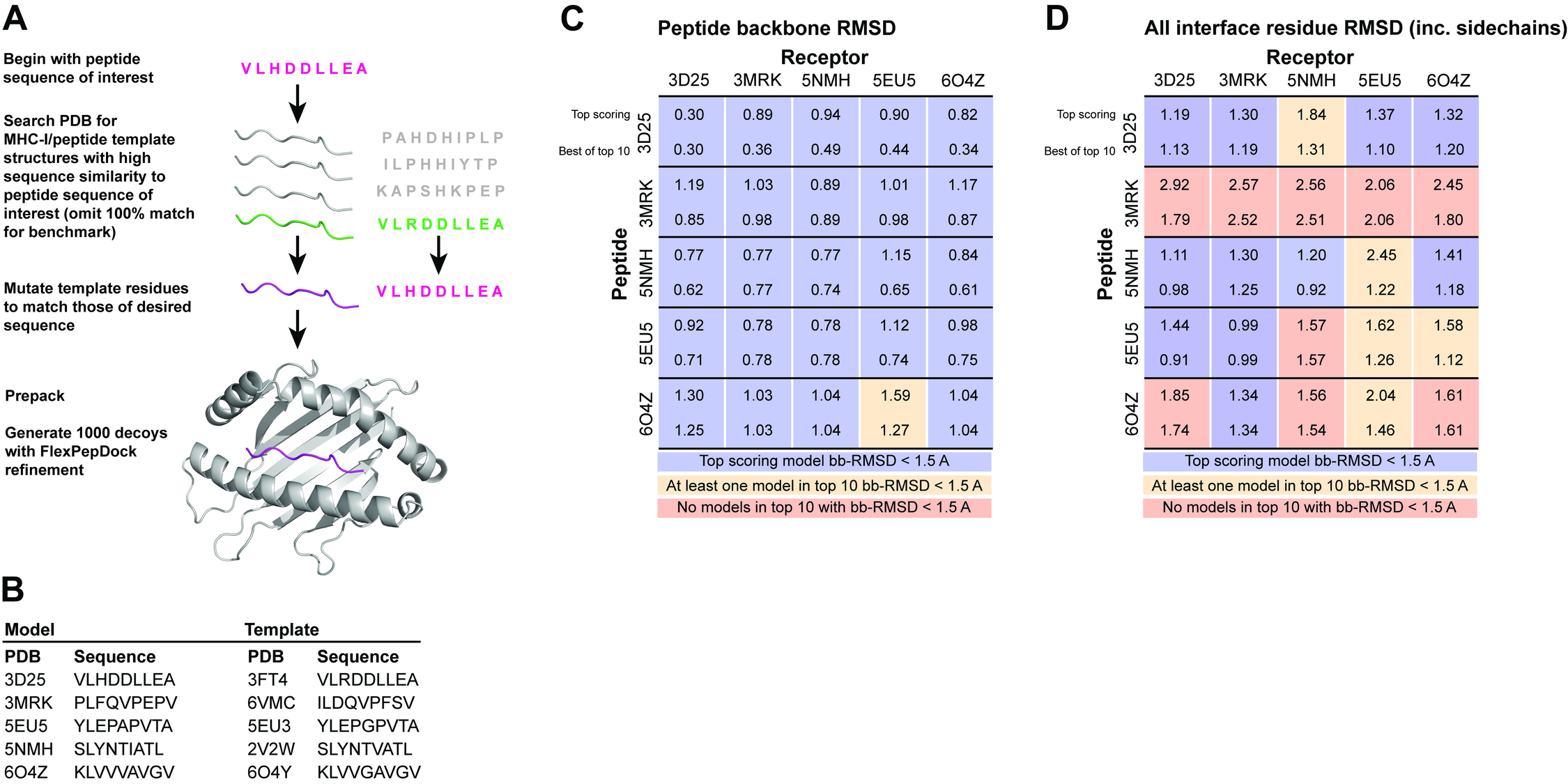

Supplement: S5 Fig — (A) Overview of template selection process for FlexPepDock Refinement. (B) Table of PDB structures used as templates and their sequences. (C) Table of peptide backbone heavy-atom RMSD values and (D) all interface residue heavy-atom RMSD values for crossdocked peptides. (TIF) [file pone.0275759.s005.tif]

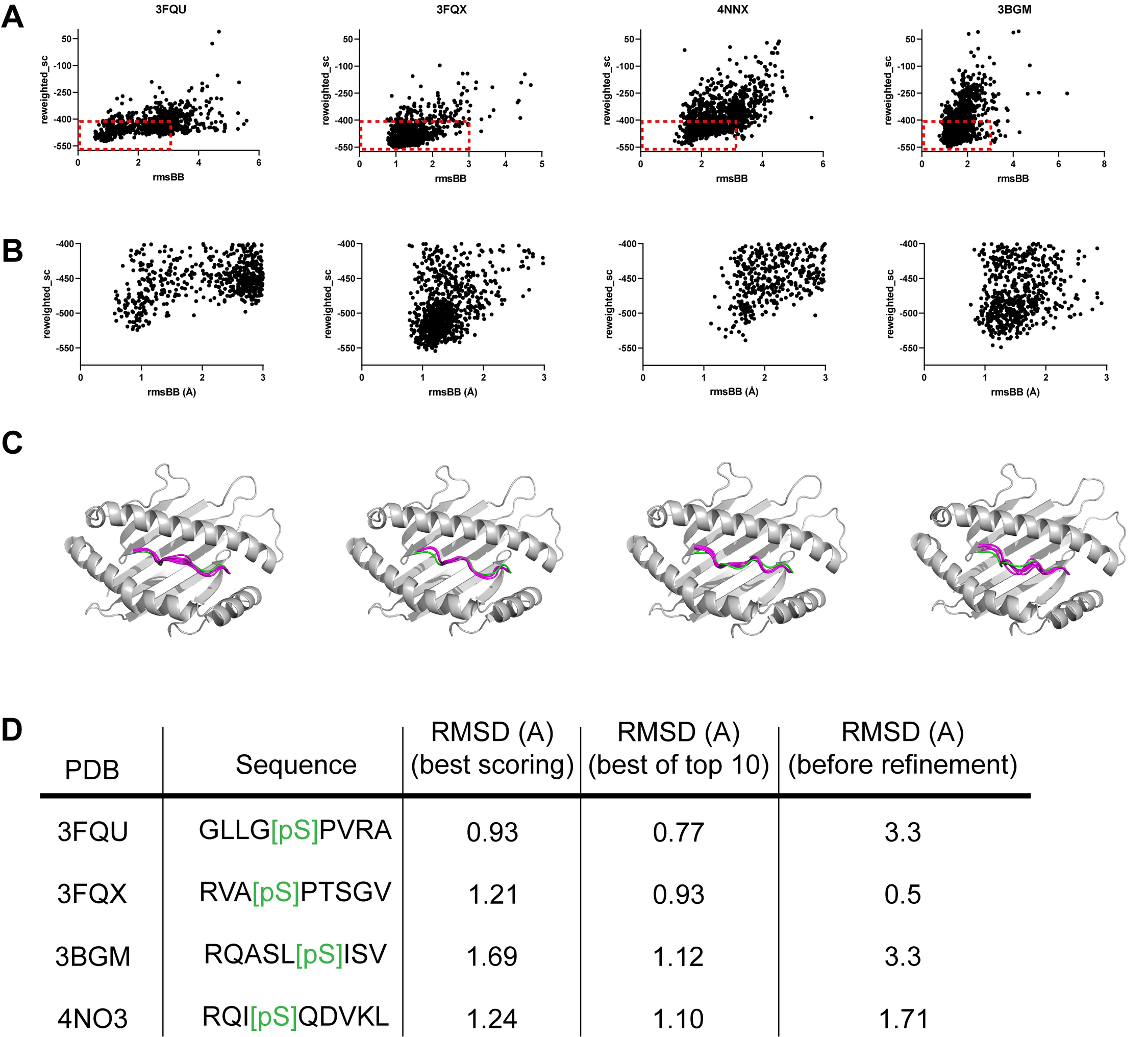

Supplement: S6 Fig — (A-B) Funnel plots illustrating the relationship between FlexPepDock Refinement reweighted score and peptide backbone RMSD. (C) Top 10 best-scoring structures generated by FlexPepDock Refinement (shown in magenta, native peptide in green). (D) Table of RMSD values for the best scoring and best of the top 10 best scoring decoys produced. (TIF) [file pone.0275759.s006.tif]
