# Supplementary material for: Rosetta FlexPepDock to predict peptide-MHC binding: An approach for non-canonical amino acids
Source: PLoS One. 2022 Dec 13;17(12):e0275759. doi: 10.1371/journal.pone.0275759 (PMC9746977; doi:10.1371/journal.pone.0275759)
Supplement: S1 File — (DOCX) [file pone.0275759.s007.docx]

### Supplemental Table 1

| Generate a custom .params file for NCAA of interest for use in Rosetta | | |
| --- | --- | --- |
| Step | Command Line | Comments |
| Set environment variables | $ROSETTA_PATH = /path/to/rosetta/main$BCL_PATH = /path/to/bcl.exe$DATABASE_PATH = /path/to/rosetta_database$BIN_PATH = ${ROSETTA_PATH}/source/bin | Path to Rosetta /mainPath to biochemical libraryPath to Rosetta databasePath to Rosetta applications |
| Create NCAA model in Pymol |  | Download .sdf files of NCAA components from pubmed.Import and merge manually in Pymol or other molecular graphics software to generate new .sdf file. Fix protons with obabel or similar for pH 7.4.FILES GENERATED:NCAA.sdf |
| Generate conformers (or rotamers) library | $BCL_PATH/bcl.exe\--natives NCAA.sdf\-max_iterations 8000\-top_models 100\-opencl Disable\-ensemble_filenames KIS_ph.sdf\-conformation_comparer RMSD 0.5\-output_rmsd_score 76_info SymmetryRMSD\-cluster\-rnd_dihedral_mutate_weight 0.0\-conformers_single_file\NCAA_conformers.sdf | This step will generate an .sdf file with 100 structures, each of which represents a rotamer (or conformer) for use as a possible side-chain conformation in Rosetta.FILES GENERATED:NCAA_conformers.sdf |
| Generate params file with molfile_to_params_polymer.py (script located at ${ROSETTA_MAIN}/demos/public/using_ncaas_protein_peptide_interface_design/HowToMakeResidueTypeParamFiles/scripts/molfile_to_params_polymer.py) | $ python molfile_to_params_polymer.py\--polymer\--name NCA\--no-pdb\NCAA_conformers.sdf | “NCA” is an arbitrary 3-letter term for our NCAA of choice.Manually designate atom numbers as described by Renfrew PD, et al. *PLoS One*. 2012;7: e32637 (see ref. [20]) in the input NCAA_conformers.sdf file.Complete documentation for generating params files with these scripts can be found at https://new.rosettacommons.org/docs/latest/rosetta_basics/non_protein_residues/Noncanonical-Amino-Acids.FILES GENERATED:NCAA.params |
| Generate conformers (or rotamers) library in .pdb format with same atom names as .params file (script located at ${ROSETTA_MAIN}/source/scripts/python/public/molefile_to_params.py) | $ python molfile_to_params.py\-n NCA\-p NCA\--conformers_in_one_file\NCAA_conformers.sdf | The location of NCAA_conformers.pdb relative to NCAA.params should be indicated by a final line in NCAA.params prefixed by PDB_ROTAMERS (see <https://new.rosettacommons.org/docs/latest/rosetta_basics/file_types/Residue-Params-file> for additional details) |
|  |  | FILES GENERATED:NCAA_conformers.pdb |

| Prepare input files for FlexPepDock *ab-initio* | | | |
| --- | --- | --- | --- |
| Prepare peptide-receptor complex | Step | Command Line | Comments |
|  | Prepare MHC-1 receptor model: remove HETATM and any chains not part of the main MHC-1 receptor chain (including beta-microglobulin) using clean_pdb.py (script located in $ROSETTA_PATH /tools/protein_tools/scripts/) | $ python clean_pdb.py 6g9q.pdb A | 6g9q.pdb is the MHC-1 receptor for this example, with the main chain designated as A in the pdb file. |
|  |  |  | FILES GENERATED:6g9q_A.pdb6g9q_A.fasta |
|  | Create extended peptides with NCAA using Rosetta’s BuildPeptide application | $BIN_PATH/BuildPeptide.linuxgccrelease\-database $ROSETTA_DATABASE\-in:file:fasta NCAA_peptide.fasta\-extra_res_fa NCAA.params\-out:file:o NCAA_extended.pdb | BuildPeptide requires an input fasta file with the NCAA indicated by X[NCA], the “NCA” being our arbitrary 3-letter code of choice for our NCAA. Fasta file example:>NCAA_peptideRQAX[NCA]LSISVFILES GENERATED:NCAA_extended.pdb |
|  | Manually place extended peptide near receptor binding cleft using Pymol or similar molecular graphics software |  | Receptor chain: APeptide chain: B |
|  |  |  | FILES GENERATED:NCAA_start.pdb |
|  | Prepack the starting structure to remove internal clashes by optimizing sidechains | $BIN_PATH/FlexPepDocking.linuxgccrelease\-in:file:s NCAA_start.pdb\-extra_res_fa NCAA.params\-flexpep_prepack\-ex1\-ex2aro\-nstruct 1 | Generates a single prepacked structure and score file. |
|  |  |  | FILES GENERATED:NCAA_start_0001.pdbNCAA_start_0001.sc |
|  |  |  | FILES GENERATED:NCAA_rlx.pdb |
| Generate Fragemnts Library | Generate secondary structure information for fragments | $ fragmentpicker_runss NCAA.fasta | Input fasta file should be identical to NCAA fasta file, with the X[NCA] replaced by a canonical AA close in structure to the modified AA. Example for phosphorylated serine residue is given below:>NCAARQASLSISVfragmentpicker_runss is a c-shell script for running PSI-BLAST, PSI-PRED, and JUFO9D (see ref [30]) and formatting the output files for Rosetta’s fragment picker application. This can also be done manually or with existing Rosetta tools. See https://www.rosettacommons.org/docs/latest/application_documentation/utilities/app-fragment-picker. |
|  |  |  | FILES GENERATED:NCAA.psipred_ss2NCAA.jufo_ssNCAA.checkpoint |
|  | Create fragments configuration and weights files | >file.cfg#pool_id pool_namefraction1 psipred 0.62 jufo 0.4 | See <https://www.rosettacommons.org/docs/latest/application_documentation/utilities/app-fragment-picker> for details on fragment selection weights and configuration files creation. |
|  |  | >file.wghts #score name priority weight min_allowed extras  SecondarySimilarity 350 0.5 - psipred  SecondarySimilarity 250 0.5 - jufo  RamaScore 150 1.0 - psipred  RamaScore 150 1.0 - jufo  ProfileScoreL1 200 1.0 – | FILES GENERATED:file.cfgfile.wghts |
|  | Generate fragments | $BIN_PATH/fragment_picker.linuxgccrelease\-database $DATABASE_PATH\-in:file::vall ${ROSETTA_PATH}/tools/fragment_tools/vall.jul19.2011.gz\-in::file::fasta NCAA.fasta\-in::file:checkpoint NCAA.checkpoint\-frags::ss_pred NCAA.psipred_ss2\-frags::ss_pred NCAA.jufo_ss\-frags::scoring::config file.wghts\-frags::picking::quota_config_file file.cfg\-frags::frag_sizes 5 3\ | Fragment sizes of 5 and 3-mer length were chosen for this study. Fragment sizes up to 9-mer can be used for peptides of length 10 or greater. |
|  |  |  | FILES GENERATED:frags.200.3mersfrags.200.5mers |
|  | Renumber fragments starting from last residue number in receptor (chain A in NCAA_rlx.pdb) | $ROSETTA_PATH/demos/protocol_capture/flex_pep_dock_abinitio/scripts/frags/shift.sh\frags.200.3mers 180 >frags.3mers.offset$ROSETTA_PATH/demos/protocol_capture/flex_pep_dock_abinitio/scripts/frags/shift.sh\frags.200.5mers 180 >frags.5mers.offset | For the example given, peptide residues begin at index 181 (adjust this index offset to your needs). |
|  |  |  | FILES GENERATED:frags.3mers.offsetfrags.5mers.offset |
|  | Replace the placeholder NCAA (S in our example) with X in the fragment files | sed ‘s/S/X/g’ frags.3mers.offset >NCAA.3mers.offsetsed ‘s/S/X/g’ frags.5mers.offset >NCAA.5mers.offset | FILES GENERATED:NCAA.3mers.offsetNCAA.5mers.offset |
| Constraint File | Prepare constraints file | >constraints.cst for H-2Db peptides# Peptide N-terminus binding site AtomPair CB 171A CA 1B FLAT_HARMONIC 8.6 1.0 0.5  AtomPair CB 171A CB 1B FLAT_HARMONIC 9.1 1.0 0.5  AtomPair CB 7A CA 1B FLAT_HARMONIC 7.8 1.0 0.5  AtomPair CB 7A CB 1B FLAT_HARMONIC 9.1 1.0 0.5  AtomPair CB 63A CA 1B FLAT_HARMONIC 6.3 1.0 0.5  AtomPair CB 63A CB 1B FLAT_HARMONIC 6.6 1.0 1.0  # Peptide C-terminus binding site  AtomPair CB 146A CA 9B FLAT_HARMONIC 6.4 1.0 0.5  AtomPair CB 146A CB 9B FLAT_HARMONIC 7.6 1.0 0.5  AtomPair CB 80A CA 9B FLAT_HARMONIC 5.3 1.0 0.5  AtomPair CB 80A CB 9B FLAT_HARMONIC 5.2 1.0 0.5  AtomPair CB 123A CA 9B FLAT_HARMONIC 9.0 1.0 0.5  AtomPair CB 123A CB 9B FLAT_HARMONIC 7.9 1.0 0.5  # Conserved MHC-1 residue W73  AtomPair NE1 73A CA 76A FLAT_HARMONIC 8.0 1.0 0.2  AtomPair NE1 73A CA 70A FLAT_HARMONIC 7.1 1.0 0.2  AtomPair NE1 73A CA 95A FLAT_HARMONIC 11.8 1.0 0.2  ================================================== >constraints.cst for HLA-A*02:01 peptides# Peptide N-terminus binding siteAtomPair CA 171A CA 1B FLAT_HARMONIC 9.7 1.0 0.25AtomPair CA 7A CA 1B FLAT_HARMONIC 9.2 1.0 0.25AtomPair CA 63A CA 1B FLAT_HARMONIC 6.8 1.0 0.25# Peptide C-terminus binding siteAtomPair CA 146A CA 9B FLAT_HARMONIC 7.5 1.0 0.25AtomPair CA 80A CA 9B FLAT_HARMONIC 7.1 1.0 0.25AtomPair CA 123A CA 9B FLAT_HARMONIC 10.2 1.0 0.25#W146AtomPair CH2 147A CA 73A FLAT_HARMONIC 9.7 1.0 0.25AtomPair CH2 147A CA 80A FLAT_HARMONIC 11.8 1.0 0.25AtomPair CH2 147A CA 121A FLAT_HARMONIC 8.7 1.0 0.25 | Example constraints file with N- and C-terminus binding site defined, in addition to conserved W73 position (used for crossdocking of H-2D^b^ bound peptides) or W146 position (used for HLA-A*02:01 bound peptides) |
|  |  |  | FILES GENERATED:constraints.cst |

| Run FlexPepDock *ab-initio* | | |
| --- | --- | --- |
| Step | Command Line | Comments |
| Prepare options file with path to required inputs defined | >flexpepdock.options# Define the input model-s NCAA_rlx.pdb# Define the native model for RMSD calculations-native NCAA_native.pdb# Define our params file# NCAA_conformers.pdb should be in same directory as NCAA.params – define this relationship on the last line of NCAA.params-extra_res_fa NCAA.params# Fragment files-flexPepDocking:frag3_weight 1.0-flexPepDocking:frag5_weight 0.25-frag3 NCAA.3mers.offset-flexPepDocking:frag5 NCAA.5mers.offset# Constraint file and relative constraint weights-constraints::cst_file constraints.cst-constraints:cst_fa_file constraints.cst-constraints:cst_weight 10-constraints:cst_fa_weight 10# Define the number of structures. Ideally 50,000 structures should be generated in total, with the program run in parallel on a computing cluster to reduce wall time.-nstruct 50000# General options for FlexPepDock-ex1-ex2aro-use_input_sc-lowres_abinitio-pep_refine-receptor_chain A-flexpep_score_only | Required inputs (generated in previous steps):NCAA_rlx.pdbNCAA_native.pdb [a native structure for calculating RMSD values – for purely theoretical structures, the input model will suffice]NCAA.paramsNCAA_conformers.pdbNCAA.3mers.offsetNCAA.3mers.offsetconstraints.cst |
|  |  | FILES GENERATED:flexpepdock.options |
| Run FlexPepDock *ab-initio* | $BIN_PATH/FlexPepDocking.linuxgccrelease\@flexpepdock.options\-database $DATABASE_PATH\-out:file:silent | Additional options for defining file prefixes, output file locations, scorefile preferences, etc. can be found at https://www.rosettacommons.org/docs/latest/rosetta_basics/options/options-overview |

The data generated for this manuscript has been uploaded to a publicly available repository located at https://github.com/orgs/meilerlab/repositories/FlexPepDockNCAA.

### Supplemental Table 2

| Peptide Sequence | Experimental EC50 (nM) | NetMHCI BA (nM) | Top 10 Reweighted Score | Top bb-RMSD |
| --- | --- | --- | --- | --- |
| ASNENMETM | 8 | 9.5 | -527.9 | 1.10 |
| AANENMETM | 5 | 6 | -540.0 | 1.36 |
| ACNENMETM | 100 | 133.8 | -540.2 | 1.72 |
| ADNENMETM | 700 | 2034.9 | -503.2 | 0.92 |
| AENENMETM | 4000 | 1095.6 | -519.7 | 0.80 |
| AFNENMETM | 3000 | 770.2 | -513.1 | 1.47 |
| AGNENMETM | 10 | 32.4 | -543.9 | 0.96 |
| AHNENMETM | 80 | 333.5 | -551.3 | 1.47 |
| AINENMETM | 10 | 92.2 | -541.8 | 1.47 |
| AKNENMETM | 220 | 854.6 | -543.2 | 1.19 |
| ALNENMETM | 80 | 112.9 | -535.9 | 1.04 |
| AMNENMETM | 0.4 | 16.8 | -547.0 | 1.15 |
| ANNENMETM | 15 | 90.3 | -555.9 | 1.04 |
| APNENMETM | 5000 | 1578.2 | -538.6 | 1.33 |
| AQNENMETM | 7 | 25 | -547.0 | 1.16 |
| ARNENMETM | 4000 | 2469.9 | -540.7 | 1.33 |
| ATNENMETM | 30 | 29.1 | -557.12 | 1.6 |
| AVNENMETM | 7 | 42.1 | -555.1 | 1.22 |
| AWNENMETM | 4000 | 2620.6 | -525.09 | 1.08 |
| AYNENMETM | 700 | 595.4 | -525.19 | 1.26 |

### Supplemental Table 3

|  | Non-phosphorylated | | Phosphorylated | | |  |
| --- | --- | --- | --- | --- | --- | --- |
|  |  |  |  | |  | |
| Peptide | IC50 (nm) | Reweighted Score | IC50 (nm) | Reweighted Score | |  |
| RQA[pS]LSISV | 284.5 | -537.84 | 38.5 | -554.90882 | |  |
| RVA[pS]PTSGV | 731.3 | -541.231 | 178.5 | -547.79312 | |  |
| GLLG[pS]PVRA | 218.8 | -383.216 | 168.7 | -560.3753 | |  |
| KILDRTE[pS]L | 101.5 | -526.766 | 62.4 | -539.48054 | |  |
| RLD[pS]YVRSL | 116.5 | -529.651 | 44.6 | -510.1969 | |  |
| RTF[pS]PTYGL | 408.2 | -535.748 | 32.5 | -509.70004 | |  |
| RQI[pS]QDVKL | N/A | -498.50 | N/A | -563.26 | |  |

### Supplemental Table 4

|  |  | **Lysine** | |  | **IsolG-Lysine** | |
| --- | --- | --- | --- | --- | --- | --- |
| **Replacement Position** |  | **Top 10 lowest Reweighted Score** | **bb-RMSD** |  | **Top 10 lowest Reweighted Score** | **bb-RMSD** |
| P1 (KAAANAAAL) |  | -467.22 | 1.1 |  | -454.52 | 1.5 |
| P2 (AKAANAAAL) |  | -552.76 | 1.5 |  | -542.62 | 1.6 |
| P3 (AAKANAAAL) |  | -503.47 | 1.3 |  | -366.89 | 1.2 |
| P4 (AAAKNAAAL) |  | -516.73 | 1.4 |  | -521.24 | 1.4 |
| P6 (AAAANKAAL) |  | -531.41 | 0.9 |  | -546.73 | 1.4 |
| P7 (AAAANAKAL) |  | -503.49 | 1.4 |  | -483.84 | 1.5 |
| P8 (AAAANAAKL) |  | -539.54 | 1.1 |  | -527.09 | 1.2 |
